# Should AMOC observations continue: how and why?

**DOI:** 10.1098/rsta.2022.0195

**Published:** 2023-12-11

**Authors:** E. Frajka-Williams, N. Foukal, G. Danabasoglu

**Affiliations:** ^1^ Institut für Meereskunde, Universität Hamburg, 20148 Hamburg, Germany; ^2^ National Oceanography Centre, Southampton SO14 3ZH, UK; ^3^ Woods Hole Oceanographic Institution, Woods Hole, MA 02543, USA; ^4^ Climate and Global Dynamics Laboratory, National Center for Atmospheric Research, Boulder, CO 80305, USA

**Keywords:** AMOC, overturning, ocean observations

## Abstract

The Atlantic meridional overturning circulation (AMOC) is a large-scale circulation pattern responsible for northward heat transport in the Atlantic and is associated with climate variations on a wide range of time scales. Observing the time-varying AMOC has fundamentally changed our understanding of the large-scale ocean circulation and its interaction with the climate system, as well as identified shortcomings in numerical simulations. With a wide range of gains already achieved, some now ask whether AMOC observations should continue. A measured approach is required for a future observing system that addresses identified gaps in understanding, accounts for shortcomings in observing methods and maximizes the potential to guide improvements in ocean and climate models. Here, we outline a perspective on future AMOC observing and steps that the community should consider to move forward.

This article is part of a discussion meeting issue ‘Atlantic overturning: new observations and challenges’.

## 1. Introduction

**Figure 1.**
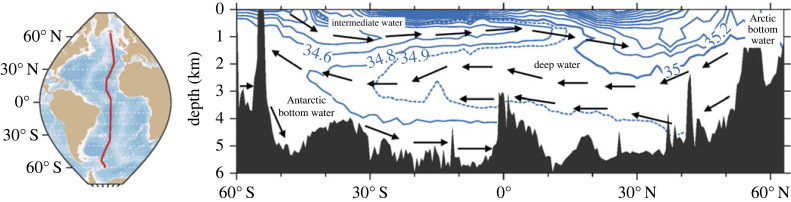
The Atlantic meridional overturning circulation (AMOC) viewed as a slice through the Atlantic from south to north [1]. Northward flowing intermediate water is found in the top 1000 m, and southward flowing water between 1000–4000 m. In the South Atlantic, there is also significant northward flowing water below 3000 m originating around Antarctica.

The Atlantic meridional overturning circulation (AMOC) is a circulation pattern or system of currents in the Atlantic which corresponds to northward flowing warm waters and southward flowing cold waters (figure 1). Unlike the Pacific, the net meridional (north–south) heat transport in the Atlantic is northward at all latitudes, whereas in the Pacific, it is poleward (and so is southward in the southern hemisphere). It has additionally received a lot of attention in the past few decades due to its hypothesized role in the rapid adjustments of global climate in past millennia. These include, for instance, iceberg discharge events like the Heinrich event 17, 500 years ago where large amounts of freshwater were released into the North Atlantic, resulting in a slowdown or shutdown of the AMOC.

Here, we will revisit our motivations for observing the AMOC, review what we have learned from making continuous AMOC observations, take stock of our current observational capability and discuss what we may expect to gain from future AMOC observations. We will conclude with a short list of recommendations for questions the community should seek to address to more fully answer the question posed here: ‘Should AMOC observations continue?’

### (a) Role of the AMOC in climate

The AMOC affects climate variability through two main mechanisms: (1) the redistribution of heat from south to north through meridional heat transport (MHT), and (2) the uptake of carbon from the atmosphere and its storage in the deep ocean. The Atlantic is distinct from all other ocean basins in that heat is transported in the northward direction across all latitudes, and studies of anthropogenic carbon content in the ocean have highlighted the northern high latitudes (northern reaches of the AMOC) as the location where the ocean amassed anthropogenic carbon [2]. We briefly highlight two examples below.

On modern time scales, the AMOC has been found to have a relationship with Atlantic multidecadal variability (AMV) [3]. This variability refers to observed fluctuations in the surface temperature averaged over the whole North Atlantic region, occurring on multidecadal time scales. The AMOC is a natural forcing mechanism that connects to the AMV due to the expected role of the ocean in long time scales. Some studies have found a correspondence between the AMOC and AMV, while others have found closer connections to other forcings such as volcanoes and aerosols (see [3], and the references therein). If the AMOC and AMV are linked, e.g. if a strengthening (weakening) of the AMOC causes an increase (decrease) in the AMV index, then associated climatic changes like drought in the Sahel and changes in hurricane frequency and intensity can also be traced back to the AMOC circulation.

On paleo-time scales, the shutdown of the AMOC has been linked to significant climate reorganization on a global scale following iceberg discharge events [4]. The AMOC has received significant attention due to this potential to shut down, reducing the northward heat transport and changing heat distribution in the climate system. An AMOC schematic view has been developed to explain paleo-time scale changes and highlights the link between northern high latitude convection (deep ocean mixing) and the downward or sinking branch of the AMOC. Stommel developed a two-box model that demonstrated nonlinearity in the system, where recovery of the AMOC back to its pre-disturbed state was more challenging than pushing it into a new state [5]. Therefore, if the AMOC shuts down, remediation efforts such as carbon capture and storage would need to overshoot to recover the AMOC. It is important to note that this view of the AMOC expects a millennial time scale of AMOC variability (with changes separated by O(1000 years)) and that the AMOC itself is viewed as a basin-wide circulation pattern (with a nominal spatial scale of O(1000 km)).

These examples highlight just two major time scales where the AMOC is expected to play a role in climate, and for which it has been a topic of intense study. These investigations have, in the past two decades, been supported by observing arrays which provided—for the first time—estimates of the continuously varying large-scale overturning circulation [6].

### (b) Value of AMOC observations

AMOC observations spanning latitudes have yielded stunning gains in understanding in a relatively short span of time (since the early 2000s). From where we started two decades ago, these gains in understanding have repeatedly called into question our fundamental notion of what the large-scale ocean circulation is. Reviews of these gains in understanding can be found in [7]. We reiterate a subset of the key findings here, but also note that the direct observations have inspired a range of numerical and theoretical advances that are further reviewed in a collection [6].

AMOC observations have confirmed that the overturning is the circulation pattern responsible for the climatically important feature of the Atlantic: the net northward heat transport across all latitudes. We have further identified that the overturning transport plays a leading role in the basin-scale redistribution of salt, carbon and nutrients. However, we have also discovered that the conceptual picture of the AMOC as ‘a slowly-varying circulation driven by convection (sinking) at high latitudes or diffuse basin-scale upwelling by mixing’ does not explain the observed variability of the AMOC. Direct observations have demonstrated that the AMOC varies on all observed time scales (from days to decades) and that the variability differs between latitudes [8]. Much of the variability of the AMOC in the subtropics can be traced to local or regional fluctuations in atmospheric wind patterns (both direct Ekman forcing, and basin-wide adjustment to wind-stress curl patterns) [9]. With this new understanding of the AMOC as a phenomenon that varies between latitudes, we have been forced to reconsider how we think about long-term proxies of change (North Atlantic sea surface temperatures or ocean heat content) and how they can be used to understand long-time-scale, basin- or global-scale past changes. The observations have further indicated that convection appears to play little role in the overturning strength in the subpolar North Atlantic [10], but rather watermass transformation east of Greenland is responsible for the overturning strength in the subpolar North Atlantic.

These findings are in contradiction with modelled variability of the AMOC, where the large amplitude of observed variability is outside the range of variability in simulated climate models, and where the modelled AMOC strength appears strongly related to the intensity of convection in the Labrador Sea [10]. As a consequence of the fundamental disagreement between observations and model results, confidence in our understanding of past AMOC variability and future evolution was downgraded in the latest IPCC report [11]. From the combination of direct observations and numerical models, we have learned that decadal predictions are improved when the AMOC is initialized with appropriate strength [12–14], and further that the AMOC measurements provide a valuable integrative benchmark for simulations which may shed light on whether simulations capture the net effects of key processes (e.g. overflows, entrainment, watermass transformation) [15]. Some of this is through understanding whether simulated AMOC transports have the right depth structure [16,17] or slope of the heat transport-to-volume transport relationship [18,19]. To date, efforts to directly assimilate transport observations have hit a number of roadblocks [20–22].

Based on our expectation of the role of the AMOC in climate (redistribution of heat and other properties), and the benefits realized from AMOC observations to date, we can answer the question in the title of the article in the affirmative: yes, AMOC observations should continue. The more nuanced question is in addressing how those observations should be made to fill existing and new gaps in our understanding of the phenomenon of the AMOC and to maintain or enhance benefits of the observations including to modelling efforts.

## 2. Observing the AMOC

Before considering the future of AMOC observations, we provide an overview of how large-scale, time-variable ocean transports are measured at present. We refer the reader to the recent reviews of AMOC observing [8,23], but reiterate here the essential details of the transport calculations needed to consider future measurement requirements.

### (a) AMOC definitions

In contrast to the schematic view of the AMOC as a large-scale (global-scale) circulation system, the AMOC is derived from the overturning streamfunction Ψ(y,z,t)

2.1 $$\Psi (z,t)=\int_{z}^{0}\int_{x_{w}}^{x_{e}}v(x,z^{\prime },t)\;dx\;dz^{\prime },$$

where the transport streamfunction is the zonally integrated, vertically accumulated meridional velocities v with the limits of integration at the western and eastern boundaries of a transbasin section, $x_{w}$ and $x_{e}$, respectively, and z defined negative downwards. Since there will be no flow below the seabed, the value of the streamfunction starts at zero at the seabed. Assumptions at different arrays may further require the streamfunction to be zero at the sea surface (e.g. at RAPID, no net throughflow is allowed by the calculation, so that Ψ(z=0,t)=0. This then gives us the overturning at a given latitude,

2.2 $$MOC_{z}(t)=\max_{z}\Psi (z,t).$$

The definition can, and perhaps should, be used in density-space rather than depth-space, whereupon the streamfunction is

2.3 $$MOC_{\sigma }(t)=\sum_{\sigma_{\min}}^{\sigma_{\max}}T(\sigma^{\prime },t)\;d\sigma^{\prime },$$

where T is northward transport in Sv associated with each density and $MOC_{\sigma }(y,t)$ is now a function of density σ (following [24,25]). We also refer the reader to the recent article by Waldman *et al.* [26] which, for the streamfunction in depth-space, usefully reframes the decomposition of the overturning into component parts.

Calculating the AMOC thus requires knowledge of meridional currents v at each longitude (or x position) and depth along a closed section spanning the full-width of the Atlantic. In the case of the AMOC in density space, it further requires a full-basin sections of temperature and salinity to correctly assign local meridional velocities to their associated density class.

### (b) Deriving velocity from geostrophy

While there are observational methods to measure currents directly, it would be prohibitively expensive to install current meters spanning large basin-widths (e.g. 6000 km at $26^{\circ }N$). Instead, we rely on the balances from the equations of motion—between velocity and pressure gradients on a rotating Earth (geostrophic balance) and between velocity and wind stress at the sea surface (Ekman transport) [26]. In this way, we can estimate ocean currents over large distances without the need for direct current measurements. However, direct current observations in narrow boundary currents are also used (e.g. in western boundary currents from moorings or the Florida Straits cable measurements) and can provide more precise measurements in regions of strong flow with large gradients [27].

If absolute pressure were known directly, then the zonally averaged meridional (geostrophic) velocity could be computed directly at a line of latitude ($y_{0}$) as follows:

2.4 $$v_{g}=\frac{g}{f}\frac{\partial p}{\partial x},$$

where $v_{g}(z,t)$ is the geostrophic velocity in the north–south direction, g is the gravitational acceleration, $f(y_{0})$ is the Coriolis frequency, and p(x,z,t) is pressure. Once we invoke geostrophy to estimate velocity in equation (2.4), we assume a constant f. If pressure along a line in x and y were known, then velocity could be calculated from geostrophy using local gradients in pressure and a local f(y) which varies with latitude. In practice, when applying equation (2.4) or thermal wind (below) to moored observations spanning the Atlantic, the horizontal separation between measurements of pressure (or seawater density) can be large, and the estimate becomes

2.5 $$\bar{v_{g}}=\frac{g}{f}\frac{\Delta p}{\Delta x}=\frac{g}{f}\frac{p_{\mathrm{east}}-p_{\mathrm{west}}}{x_{\mathrm{east}}-x_{\mathrm{west}}},$$

where $\bar{v_{g}}$ is the average meridional velocity between the two points in x where the variations in pressure between points $x_{\mathrm{east}}$ and $x_{\mathrm{west}}$ are not known. If the horizontal separation in y or latitude is also large, then a fixed f must be chosen.^1^

In practice, absolute pressure is challenging to measure *in situ* due to drift in pressures sensors [29,30]. Instead, we use vertical profiles of seawater density and rely on hydrostatic pressure to relate pressure at a depth to the weight of water above it. This allows us to estimate the vertical shear of meridional (geostrophic) velocity from zonal gradients in seawater density as follows:

2.6 $$f\frac{\partial v_{g}}{\partial z}=-\frac{g}{\rho_{0}}\frac{\partial \rho }{\partial x},$$

where ρ(x,z,t) is seawater density. This second version of geostrophic balance, called *thermal wind*, only gives us an estimate of the vertical shear (∂/∂z) of velocity and requires an integration constant in order to derive absolute geostrophic velocities.

2.7 $$v_{g}(z)=-\int_{-h}^{z}\frac{g}{\rho_{0}f}\frac{\partial \rho }{\partial x}\;dz+v_{\mathrm{ref}}.$$

Note that $v_{\mathrm{ref}}$ is only a constant with respect to a specified depth h, but can vary with time, latitude–longitude position, and the choice of the limits of the vertical integration. In oceanography, this constant is referred to as the (geostrophic) velocity at the reference level ($v_{\mathrm{ref}}$), where it represents the zonally averaged velocity at fixed depth between the two locations in x. It is called the level-of-no-motion if the constant of integration is set to 0, or the level-of-known-motion if the constant of integration is set to a non-zero value.

### (c) *In situ* AMOC volume transport measurements

Before the 2000s, the AMOC was measured from hydrographic sections which involved lowering a conductivity-temperature-depth (CTD) device from a ship to measure the vertical profile of seawater density ρ(z) at different points along a line of latitude. By calculating the vertical shear of meridional velocity ∂v/∂z between two successive profiles (in the case of hydrographic sections, these are relatively close together or O(100 km) apart), the thermal wind relationship (equation 2.6) can be used to estimate AMOC transport. This involves integrating the meridional component of the velocity profile and vertically accumulating it as in equation (2.2) to compute the overturning strength in Sv (Sverdrups, a unit of volume transport equal to one million cubic meters per second).

**Figure 2.**
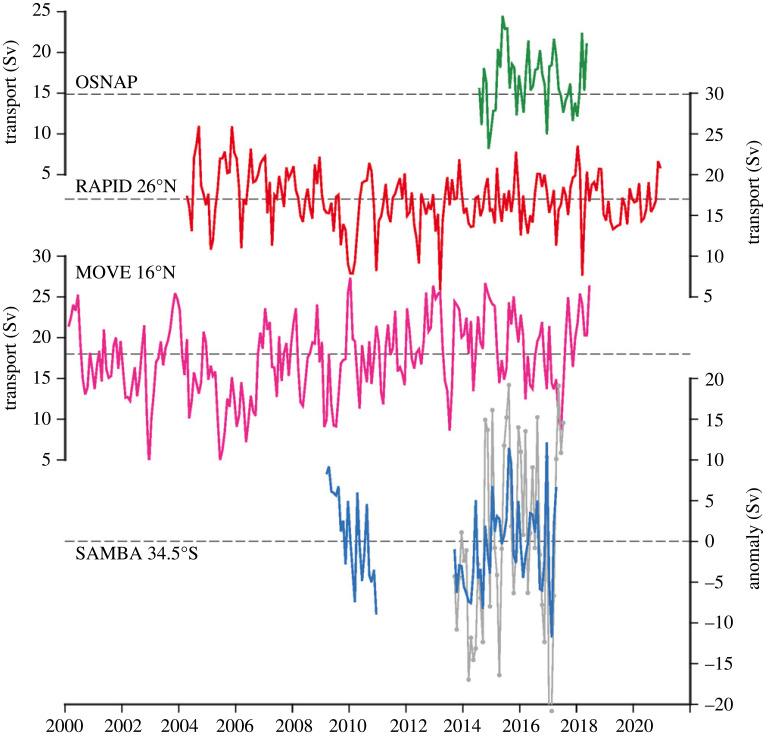
Monthly values of AMOC transport from four observing arrays, updated from [8]: OSNAP (green), RAPID $26^{\circ }N$ (red), MOVE $16^{\circ }N$ (magenta) and SAMBA $34.5^{\circ }S$ (blue/grey). For SAMBA, two estimates are shown where the blue values are from [31] and the grey values from [32].

The AMOC has been observed using continuous observing techniques (e.g. moored instruments) since 2001 (partial basin array) and since 2004 (full basin array), summarized in table 1 and figure 2. A review of the observing efforts can be found in [8]. The general principles that underly all the observing arrays are the application of the thermal wind relationship (equation (2.6)), computation of Ekman transport, and a subset of the arrays also directly observing meridional velocities near continental slopes. Here, we will only highlight some of the overall conclusions and open questions.

**Table 1.** AMOC observing arrays, with their nominal operational dates, name and approximate latitude (or range) and observations used.

date	name	latitude	instruments
2014–present	OSNAP array	$53\text{--}60^{\circ }N$	Moorings, Argo & gliders
2013–2017	NOAC	$47^{\circ }N$	Moorings, PIES
2004–present	RAPID/MOCHA/WBTS array	$26^{\circ }N$	Moorings
2001–present	MOVE array	$16^{\circ }N$	Moorings
2013–present	TSAA/TRACOS	$11^{\circ }S$	Moorings, PIES
2009–2011, 2013–present	SAMBA	$34.5^{\circ }S$	Moorings, PIES

At the time when mooring arrays were installed, it was a novel idea to use moored measurements to estimate large-scale transport variability (i.e. transports integrated over thousands of kilometres). The benefits of the approach were quickly apparent after the first year and then first few years of the observations, when the measurements revealed striking variability at seasonal time scales [33] as well as on time scales as short as 10 days. Some criticism ensued, most prominently concerning the role of eddies and small-scale noise drowning out any large-scale signal [34]. But the success of the RAPID program has largely assuaged those criticisms [35], demonstrating that the transbasin mooring approach can indeed work.

### (d) Sources of uncertainty in AMOC observations

While the equations used are simple, measuring the AMOC is not straightforward and carries with it a number of potential sources of error and uncertainty. The true overturning strength through a section comes from the velocity normal to the section at every point in depth and along the section. The velocities would then be integrated as in equation (2.1) to separate the net northward flow from the net southward flow. In practice, the calculated strength of the AMOC is an approximation of the true value: we cannot measure velocity everywhere and rely primarily on sparse measurements of ocean temperature, salinity and pressure to estimate velocity. The measurements may have errors due to instrument calibration errors or drift. In addition, there are structural uncertainties arising from limitations in the methods used to estimate the AMOC. These include methodological choices such as how to fill data gaps (in time and space) and how to apply the choice of reference level.

The concept of uncertainty refers to a measure of a range of values within which the true value of the quantity is expected to lie. Uncertainty takes into account all of the potential sources of error and provides a quantitative measure of how confident we can be in the measured value. The term also applies to the uncertainty on a mean which reflects the impact of variability on our ability to estimate the mean value of a quantity, such as the annual average of the AMOC. For a quantity such as the AMOC with strong high-frequency fluctuations, the uncertainty on the mean becomes relevant when we consider estimating the AMOC through methods that subsample in time (e.g. Argo profiling floats) compared to continuous measurements from moorings.

**Figure 3.**
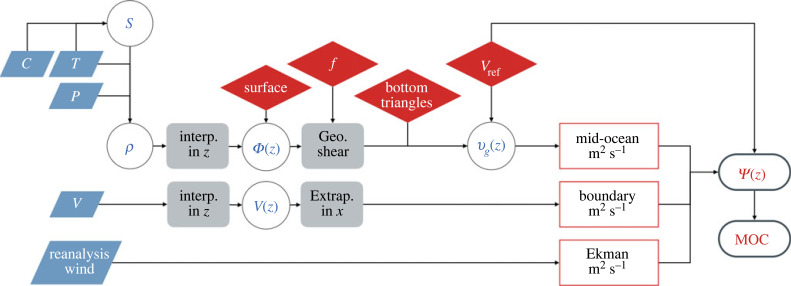
Flowchart schematic showing the measurements, calculations and choices that are made to estimate the AMOC at $26^{\circ }N$. Blue parallelograms are measured variables (e.g. temperature T, conductivity C, pressure P, velocity V and also including reanalysis winds); white circles are derived quantities (e.g. salinity S, density ρ, dynamic height Φ(z), velocity profiles V(z) and absolute geostrophic velocities $v_{g}(z)$). Calculations are in grey (e.g. interpolating discrete measurements onto a regularly spaced vertical profile, calculating geostrophic shear between profiles of dynamic height, or extrapolating from moorings to the continental slope), while methodological choices are in red diamonds (e.g. the choice of filling data in the near-surface layer, choice of latitude for f, how to fill bottom triangles, and calculating the reference level velocity $v_{\mathrm{ref}}$). The component transports in units of transport-per-unit-depth ($m^{2}\;s^{-1}$ or Sv m^−1^) are in red rectangles (mid-ocean calculated from dynamic height, boundary currents from direct velocity measurements and surface Ekman transport), while the AMOC transports (streamfunction Ψ(z) and maximum MOC) are in ovals. The arrows show the flow of information. Measured quantities may have measurement errors associated with them, while methodological choices can introduce differences between AMOC array estimates.

**Figure 4.**
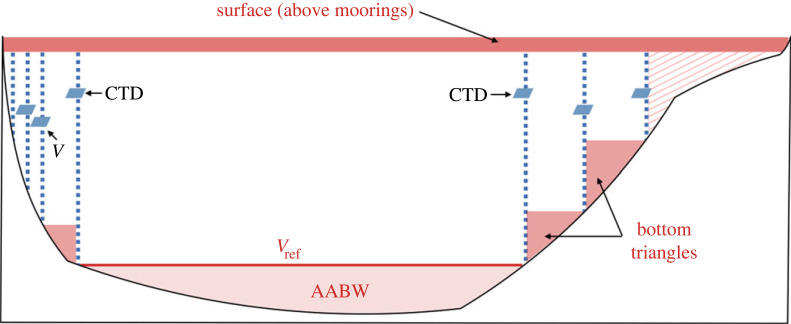
Diagram of a section across the Atlantic showing where (geographically) measurement and methodological uncertainties enter into AMOC calculations. Moorings are shown in blue dashed lines, where boundary moorings measuring velocity are at the left/western edge while the other moorings have CTD measurements. The methodological choices of what to do about unmeasured regions are in red shading including the surface (top ∼50 m above the tops of sub-surface moorings), the bottom triangles between deep moorings and the continental slope, the abyssal transports (in the case of $26^{\circ }N$, this is the Antarctic Bottom Water (AABW)) and the continental shelf (in red hatching). The choice of a reference level velocity is indicated here as $v_{\mathrm{ref}}$ or the reference level velocity. For RAPID, this is using a deep level (4820 dbar), but it could be at an intermediate depth (in the case of SAMBA $34.5^{\circ }S$) or at the sea surface (as for OSNAP).

Figure 3 illustrates the sources of uncertainties and errors in the AMOC calculation—represented with a flow chart—based on the example of the RAPID $26^{\circ }N$, while figure 4 shows where, geographically, these sources occur. The blue filled parallelograms in figure 3 represent where measurement error, such as sensor inaccuracy, enters the calculation. This include errors on temperature and salinity measurements due to instrument drift or calibration offsets. Error in temperature measurements is typically small, but errors in salinity can be large enough to influence transport measurements. Current metre error is relatively small, in part also because each measurement is applied to a local area (whereas salinity errors on geostrophy can be multiplied by a basin width of 6000 km). The flowchart (figure 3) also includes reanalysis winds; however, errors in these estimates are from reanalysis models rather than measured quantities. They will not be considered in detail here, except to recommend that a common reanalysis product and method of estimating wind stress from winds should be used for multi-latitudinal comparisons to avoid added uncertainty—including potential biases that result in differences in the mean contribution of Ekman to the AMOC calculation—due to a different choice of the reanalysis wind product [32,36].^2^

Methodological choices and calculations are shown in grey rectangles and red diamonds (see figure 3). The main methodological choices include how to fill gaps in unmeasured regions which include near-surface and bottom triangles, and the choice of reference level velocity ($v_{\mathrm{ref}}$). There are also choices made for the value of f both for the geostrophic shear calculation (§2b) and the estimate of Ekman transport, but these will not be discussed further here. In addition, there are choices made for how to interpolate between instruments in the vertical (the leftmost grey boxes) which can include vertically interpolating over spans of up to 500 m in the deep ocean. The method used to interpolate between instruments may vary between arrays—at $26^{\circ }N$, the method is following Johns et al. [38]—however, the error is not expected to dominate the uncertainty. It can, however, confound comparisons between dynamic height estimates from moorings and from altimetry [39] and so may warrant a closer look. It will not, however, be discussed further here.

#### (i) Salinity measurement accuracy

McCarthy *et al.* [40] highlight the importance of salinity accuracy for the AMOC volume transport estimates at $26^{\circ }N$. At 26°N, a bias between salinity profiles at the east and west of the basin of 0.003 [psu] would result in a 0.7 Sv error in the MOC transport. Such a bias could arise, for instance, if the ship-based CTDs used to calibrate the moored microCATs differed by 0.003 [psu], a difference that would then be applied to the microCAT sensors before calculating transports. For AMOC arrays where moored instruments in the east vs the west are calibrated on different cruises (e.g. RAPID $26^{\circ }N$ and OSNAP), such an offset might arise between CTDs on different ships if they are out-of-calibration or only a small number of salinity samples are taken to check the ship-based CTD calibration. While accepted practice also includes using standard seawater [41]—bottled water that is produced in laboratories and used at sea to check the calibration on the ship-based CTD—small differences can also arise between standard seawater products.

It might be possible to independently check the derived or measured temperatures and salinities using alternate methods, e.g. sea surface height anomalies which could be compared to steric height calculated from a tall (sub-surface to sea bed) mooring measuring temperature and salinity. A simple back-of-the-envelope calculation gives bounds on these estimates. Applying a bias (offset) of salinity of 0.003 to a subtropical Atlantic profile results in a steric height offset of about 1 cm at the sea surface (integrated from 4820 dbar). These numbers are small and so may be within measurement limitations when considering that moorings subsample in the vertical and typically do not measure properties in the top 50 m [39], but may provide some method to independently check for consistency.

Additional salinity (and temperature) uncertainty arises for the methods that do not use CTDs, but instead use inverted echo sounders to infer properties from travel time (e.g. SAMBA and NOAC). These approaches typically rely on a fixed-in-time relationship between travel time and profile to temperature and salinity [31].

#### (ii) Reference level choices

Any transport calculation based on thermal wind (measurements of seawater density) will *require* a treatment of the reference level constant of integration in equation (2.7). There is no way around this in the methodology for transbasin transport measurements from seawater density. (Note the emphasis here on density. If instead pressure were measurable, then we could invoke instead equation (2.4) and directly estimate geostrophic velocities rather than geostrophic shear.) Historically, this method has been applied to hydrographic sections where a level-of-no-motion $\left(v_{g}(-h)=0\right)$ is applied at some value of h where meridional flow at this depth is set to zero. For instance, for historical hydrographic sections across $24.5^{\circ }N$, a level-of-no-motion was chosen to be at 1000 m in the western boundary, and 3200 dbar or 4000 dbar in the region east of the western boundary [42,43]. While various reasons can be given to justify the treatment of reference level velocities, these reasons tend to be derived more from expectations about the circulation (e.g. for $24\text{--}26^{\circ }N,\;$ we expect that the circulation in the deep eastern basin is small) rather than observable quantities.

The choice of the integration constant varies between the arrays, with for example

— RAPID $26^{\circ }N$ using deep level of no motion at 4820 dbar, then applying a hypsometric compensation to satisfy a zero net mass transport assumption (i.e. computing a time-varying reference level velocity to ensure net transport across the section is zero) [40],
— MOVE $16^{\circ }N$ using a level-of-no-motion at 4950 m [44,45],
— OSNAP using a level-of-known-motion at the surface which is given from time-mean geostrophic surface currents from altimetry, then applying a compensation transport [8,24],
— SAMBA $34.5^{\circ }S$ using a level-of-known-motion referencing velocities at 1350 [31] or 1500 m [32] to model output, and publishing only transport anomalies rather than mean transport.

These choices are made based on expectations and out of necessity, when alternate methods are not available to check the choice [46].

Reference level choices have caused a complication in comparing AMOC volume transport estimates between latitudes. At $26^{\circ }N$, an assumption of zero net mass transport across the section (water going north must be balanced by water coming south) creates a ‘loop’ in the calculation methodology (figure 3). The reference velocity is calculated by summing all measured/estimated transports across the section and selecting (for each point in time) a velocity that closes the streamfunction, i.e. so that the streamfunction (2.1) starts and ends at zero. This gives a time-variable velocity $v_{\mathrm{ref}}$ applied uniformly across the section in x and z, with a large implied transport magnitude (about 10 Sv) and non-negligible low-frequency variability [47]. Without applying the reference level velocity (called ‘compensation transport’), the estimated trend in $26^{\circ }N$ AMOC transports over an 8-year period would have the opposite sign as the trend calculated after applying the compensation. Comparing AMOC estimates from the RAPID $26^{\circ }N$ and MOVE $16^{\circ }N$ arrays revealed opposing sign tendencies in the period 2004–2015, despite similar measured changes in properties at the Atlantic’s western boundary (increasing shear between 1200 and 4000 dbar). Further tests of the RAPID and MOVE methodologies have suggested that the difference is due to the reference level method [45], highlighting that it is critical to address this source of structural uncertainty in AMOC observing methodologies. The choice of a uniform compensation velocity is the simplest choice that can be made, and in the absence of further information is the choice used at $26^{\circ }N$; this may, however, have consequences and associated uncertainties in the calculation of AMOC heat and freshwater transports since in reality the compensation velocity may depend on x as well, which could also change the vertical structure of the compensation transport.

#### (iii) Filling measurement gaps

Unmeasured areas occur outside of the regions spanned by moorings or other *in situ* assets. Unmeasured areas include shallow regions above the top instrument on a sub-surface mooring, the so-called bottom triangles between deep moorings and the continental slope, and shallow shelf regions (figure 4). The unmeasured areas introduce error to a transport calculation, where the magnitude of the error depends on how large and variable the unmeasured transports are.

Most oceanographic moorings used are sub-surface (i.e. their top instrument is some 10s of meters to 100 m below the sea surface); this leaves a gap in measurements near the surface. At $26^{\circ }N$, the choice of infilling in the near-surface layer was found to miss the true seasonality of transports in the upper ocean, first identified in [48]. A new seasonal extrapolation method was introduced, when AMOC transports were recalculated, they were 0.4 Sv lower in the mean than when using the previous method, and 1.0 Sv lower in late summer (modifying the computed seasonal cycle relative to the previous methodology) [40]. The top 50 m is the most variable part of the water column due to interactions with the atmosphere, thus gap filling based on a seasonal climatology will underestimate the true variance. This lack of observations in the upper 50 m also likely plays an outsized role in the meridional heat and freshwater transport (MHT, MFT) estimates due to strong stratification near the surface and surface-intensified currents.

For bottom triangles, in the case of $26^{\circ }N$, the deep flows in the eastern bottom triangles are expected to be small so that the missing variability contributes little to the total overturning. However, had the unmeasured bottom triangles been collocated with the deep western boundary current (strong mean transport and strong variability [49]), they would certainly degrade the transport estimate. At OSNAP, the moorings are sited across steep bathymetry to minimize the area of bottom triangles. However, the strong, bottom intensified deep western boundary current (DWBC) causes the transports to vary widely depending on how these regions are treated.

Shallow shelves can also represent a significant source of uncertainty. In the case of OSNAP, the shallow shelves—across the eastern and western boundaries as well as across Greenland—present a potential issue due to difficulties in measuring there ice and icebergs, strong currents and strong variations in properties, including freshwater. For the Labrador shelf, a single mooring is used to represent the transport variability across a 200 km wide shelf. The coastal current in this region likely transports up to 2 Sv of very fresh water and may be an important unmeasured part of the total transbasin MFT. Where data are missing (by design or due to losses), they are infilled using proxy estimates from nearby moorings or climatological data.

#### (iv) Aliasing of high-frequency variability

The issue of aliasing (uncertainty on the mean) is not represented in figure 3. Rather, this is an issue which results from how frequently measurements of the AMOC strength are made. Given our fundamental expectations about the AMOC’s role in climate, and what we have now learned about AMOC variability from direct observations, we expect AMOC variations to influence climate on millennial time scales [50], centennial time scales [11], decadal time scales [3], interannual time scales [51] and perhaps on sub-seasonal to seasonal time scales [52]. It is unclear whether shorter (5-day) time scale variations are important [53]. Due to the long-time scale expectations, previous estimates of the AMOC were made from decadal hydrographic surveys [54].

However, the measurements used in calculating AMOC variability vary on time scales as short as sub-daily. The moored observations at boundaries are typically configured to measure every 30 minutes to 4 hours, depending on battery capacity and power consumption. These hourly measurements are low-pass filtered to suppress shorter time scales (e.g. tidal and near-inertial waves). At $26^{\circ }N$, this is accomplished by filtering individual instrument records using a 2-day sixth-order Butterworth low-pass filter [28]. Without applying such a filter, it is possible that sub-daily fluctuations of up to 50 Sv, such as those simulated in an ocean-only model [55], would appear. The data are then further 10-day low-pass filtered before applying compensation transports [46] to arrive at the time series provided by the array. This represents successive averages so that the time series is representative of a (approximately) 10-day average of the circulation.

If high-frequency fluctuations are not resolved by the measurements, i.e. if the measurements ‘sub-sample’ the circulation strength with reduced temporal resolution, then high-frequency fluctuations will project onto low-frequency variability. In comparison to the successive filtering applied at $26^{\circ }N$, an individual Argo float makes a single profile measurement every ∼10 days. Even if there were an Argo float profile at the western boundary of the Atlantic every 10 days, an AMOC estimate derived from these profiles would not be equivalent to the moored measurements where each 10-day value represents a 10-day average (rather than an instantaneous measurement once every 10 days). If the signal has substantial tidal (e.g. 12.4 hours) periodicity and subsampled every 10 days, these high-frequency variations would be aliased onto low frequencies in the subsampled record and may be interpreted as spurious variability or trends.

An example of aliasing on longer time scales was demonstrated at $25^{\circ }N$, where the overturning transport was estimated from five hydrographic sections between 1957 and 2004 [54], essentially subsampling the variability to 5 points in time over a 50-year period. The computed strength of the overturning from these hydrographic sections declined nearly monotonically from 23 to 15 Sv over a ∼50-year period from the five sections. It was later determined from the array-based observations at $26^{\circ }N$ that much of the apparent decline could be explained by the seasonal cycle of the AMOC [33]. Given the observed high-frequency, large amplitude fluctuations in AMOC variability, the issue of aliasing *must* be considered when designing an observing system. Measuring at higher frequencies allows the possibility of averaging to reduce the risk of aliasing.

### (e) Recent developments in ocean observing

#### (i) Autonomous platforms: gliders and Argo profiling floats

Since the installation of the RAPID $26^{\circ }N$ array in 2004, great strides have been made in ocean observing from autonomous platforms [56,57]. These include particularly the global Argo profiling float array—which makes about 3000 profiles of temperature and salinity in the top 2000 m of the ocean every 10 days—and long endurance autonomous vehicles or gliders (measuring in the top 1000 m). Given our current method of measuring overturning using seawater density, either of these platforms could be used to make vertical profiles of temperature and salinity, and offer some advantages to our current methodology by sampling the upper 50 m above the moorings. Argo floats will not necessarily help with the gap on the upper continental slope and shelves, however, as they typically have a deeper parking depth (∼1000 m) and measurements in shallower water depths are infrequent.

In isolation, profiling floats and gliders would make a poor substitute for the high-time resolution measurements from moorings. In particular, while there are a large number of Argo float profiles every day, these are distributed throughout the world’s oceans. As discussed above, at any one location (e.g. near a boundary where there is an existing boundary current array), there may only be a handful of profiles. For example, in the case of the western boundary of the RAPID array, there were roughly 2 profiles/month in the direct vicinity of the boundary. Given the known high-frequency variability (on time scales of hours to months), this would mean that the infrequent Argo float profiles would alias the transport measurements. In addition, the estimated accuracy of salinity in delayed mode Argo data can include biases on individual floats at a level of 0.01 [58]. Given that a bias of 0.003 in salinity can result in a transport bias of 0.7 Sv, using Argo profiles could result in an error of 2.3 Sv, or 15% of the mean. If the error in the required region is random over multiple floats, then averaging over larger numbers of Argo floats might reduce the error; the combined effect of random errors and potential aliasing due to subsampling should be tested.

Autonomous underwater gliders have the potential to solve the issue of too few profiles (compared to Argo profiling floats) due to their ability to be piloted. Gliders can then be piloted to maintain position and provide roughly six to eight profiles (top 1000 m) per day. However, glider procedures for calibration are not as well developed as for the Argo float array. The nominal accuracy of salinity measurements from gliders has not been rigorously quantified though it has been shown that salinity spiking is reduced when using a pumped CTD compared to an unpumped CTD [59], and errors on the order of 0.3 psu have been found and corrected [60]. Residual errors after correction should be evaluated to clarify whether residual accuracy is sufficient for large-scale transport estimation (noting however that gliders are currently limited to measuring in the top 1000 m of the water column, e.g. [61]).

#### (ii) New options for reference-level velocities: sea level and ocean bottom pressure

One of the fundamental methodological differences between current AMOC observing approaches is the treatment of the reference level velocity. However, recent developments in both *in situ* and remote sensing approaches may offer a solution to this problem. Geostrophic shear as calculated from equation (2.6) can be referenced to any level of known or measured velocity, including surface velocities which can be calculated from sea level slope as follows:

2.8 $$v_{\mathrm{surf}}=-\frac{g}{f}\frac{\partial \eta }{\partial x},$$

where η(x,y,t) is sea level, or from ocean bottom pressure as follows:

2.9 $$v_{\mathrm{bot}}=-\frac{1}{f\rho_{0}}\frac{\partial p_{bot}}{\partial x},$$

where $p_{\mathrm{bot}}$ is bottom pressure. Note that equation (2.9) assumes a flat bottom, but can be adapted for sloping bathymetry by using hydrostatic adjustment. That is to say, a deep measurement of pressure can be used to estimate the pressure at some level above it (say, 500 m higher in the water column) by removing the contribution of the weight of water (integrating measured density over the 500 m thickness).

Recent estimates of the AMOC using altimetry-based estimates of surface velocities have shown too-large variability compared to expectations from *in situ* measurements (e.g. the scale factor in [62] and OSNAP using time-mean rather than time-variable altimetry as a reference velocity [8]). The seemingly too-large fluctuations in sea surface height near mooring locations will potentially be improved when data from the new SWOT (Surface Water and Ocean Topography) altimeter, launched in December 2022, become available [63]. This is because the new SWOT mission will have ‘swath-style’ estimates of sea level variability, rather than along-track nadir measurements at 6-km resolution. The higher resolution data will make gridded altimetry products less sensitive to gridding choices [39]. There will be a number of wrinkles to iron out to introduce a new data stream to the suite of altimetry products, but the new SWOT-style altimetry offers the potential to reduce the reliance on gridding methodology.

Recent developments have also become available for *in situ* measurements of ocean bottom pressure. Typical ocean pressure sensors are subject to an exponential and linear drift, with the stated accuracy of the measurement given as a percentage of the total (i.e. the depth rating of the instrument) [29]. In the case of a bottom pressure sensor to be deployed at 5000 m, an initial accuracy of 0.01% for a Sea-Bird Electronics 26plus with quartz sensor, gives an initial accuracy of ∼50 m, while a stability of 0.02%/year means the measured values could drift by 1 m/year. Staggered-in-time deployments have been used to remove the initial exponential drift, but still require the removal of a linear drift over the remainder of the record, leaving uncertain the long-term tendency in the signal [30]. New pressure sensors designed to measure their own drift have become available. These instruments have a pressure casing within the outer pressure casing which is designed with a small internal volume at near atmospheric pressure (∼10 dbar). Within this small volume is a pressure sensor with an accuracy of 0.01% of 10 dbar or 0.1 cm. A second pressure sensor measures the ambient pressure outside the instrument (pressure in the ocean) and occasionally is ducted to measure the pressure inside the small volume. In this way, the change in the offset between the ambient pressure sensor and the sensor inside the small volume can be measured, i.e. the drift of the ambient pressure sensor is measured and can be removed from the measured signal of ambient ocean pressure. Such drift-free or self-calibrating pressure sensors could be used to estimate deep reference level velocities through equation (2.9), offering the potential to reconcile a serious methodological difference between observing arrays (§2d(ii)).

#### (iii) Multi-observational approaches

Approaches using ocean bottom pressure from satellite or *in situ* observations were summarized in previous reviews [8,23]; however, the potential of multi-observational approaches has not yet been fully realized. Several recent studies include [62,64,65]. The combination of *in situ* measurements of temperature and salinity (enabling the estimation of seawater density or dynamic height), and satellite-based measurements of sea level, and either *in situ* or satellite-based measurements of ocean bottom pressure, present additional constraints on the accuracy of individual measurements.

As just one example, velocity at some sub-surface depth $z_{1}$ can be estimated through geostrophy referenced to the surface or the bottom as follows:

2.10 $$\begin{array}{rl} v(x,y,z_{1},t) & \;=-\frac{g}{f}\left(\frac{\partial \eta }{\partial x}+\frac{1}{\rho_{0}}\frac{\partial }{\partial x}\int_{z_{1}}^{\eta }\rho (x,y,z^{\prime },t)\;dz^{\prime }\right), \end{array}$$

2.11 $$\begin{array}{rl} & \;=-\frac{1}{f\rho_{0}}\left(\frac{\partial p_{\mathrm{bot}}}{\partial x}-g\frac{\partial }{\partial x}\int_{-h}^{z_{1}}\rho (x,y,z^{\prime },t)\;dz^{\prime }\right). \end{array}$$

By combining sea level anomaly, ocean bottom pressure and dynamic height, it may be possible to constrain meridional transport estimates to better quantify residual error. Multi-observational approaches also offer the possibility to identify and correct for calibration errors between mooring deployments (e.g. an offset from one mooring period to another which may result from calibration problems) or to identify and correct for salinity offsets on autonomous platform-based measurements. Near-real time observations from satellites and autonomous platforms may additionally offer the potential to estimate the AMOC transports in near-real time (rather than delayed by 1–2 years according to the mooring servicing schedule) which could then be updated with more accurate estimates when moorings are recovered. To date, most observing system experiments have relied heavily on either Argo-based approaches [66] or mooring-based approaches [67]. It will be worth considering how blended approaches can be used to improve observing capability and/or reduce marginal costs of an AMOC observing system. However, inferences from model-based observing system experiments (OSEs) may still be limited by the model’s ability to replicate the real ocean, and so coordinated design studies using both models and observations are needed.

### (f) Summary

Existing approaches to observing the AMOC centre on mooring array-based methods, and so the above sections have outlined the methods employed by observing arrays and sources of uncertainty in those measurements. We also briefly outlined recent advances in ocean observing technologies which may be used to estimate overturning transports. Key take-home messages are as follows:

— Treatment of the geostrophic reference level can have a leading-order influence on transports, including on low-frequency (8-year) tendencies. New methods to measure sea surface height and ocean bottom pressure may offer opportunities to reconcile reference level transports.
— Salinity accuracy can have a leading-order influence on transport estimates, rendering calibration methods critical and substantiating the need for agreed best practices between moored approaches to geostrophic transport estimation. Calibrations of moored instruments can be checked upon deployment and recovery. Approaches for checking and correcting salinity measurements from autonomous platforms are not currently able to provide the required accuracy for transport estimation.
— Given the high-frequency variability of observed AMOC transports, sub-sampling of transport variability as from hydrographic sections and Argo float profiles may increase uncertainties on mean AMOC estimates above tolerance. Moorings are reliable (compared to, e.g. autonomous underwater vehicles), can measure across a wide span of depths (50–6000 m) and enable high-frequency sampling to avoid aliasing.
— Measurement gaps (e.g. near surface, on shallow continental shelves, in bottom triangles) introduce uncertainty in AMOC estimates. Methodological approaches to dealing with gaps will necessarily differ between regions, but sensitivity of transport estimates to methods should be quantified to evaluate whether resulting uncertainties are tolerable.
— Satellite altimetry still requires further evaluation to determine how to use it to measure the AMOC due to discrepancies between variability (higher in altimetry than dynamic height from moorings) at boundaries.

Note that in the aforementioned list, we have referred to ‘required accuracy’ and tolerable uncertainties. These will be discussed further in §4.

## 3. Evaluations of AMOC observing

Ocean observing system design refers to the process of deciding how to set up a system of measurements to achieve a specific outcome. It was used during the WOCE-to-GOSHIP evolution to decide whether and which hydrographic sections should be continued and at what frequency, and in planning the Argo profiling float array to decide how many floats would be needed to provide ocean data representative of variability at 3${\;}^{\circ }\times 3^{\circ }$ at 3-monthly time resolution. It requires some *a priori* knowledge about the scales at which the ocean varies to define measurement approaches. The AMOC observing arrays have been designed and subsequently re-evaluated using observing system experiments as summarized below. However, these were primarily carried out in the context of a single-latitude approach to AMOC observing, with strong tailoring towards methodological adaptations for each latitude and likely sensitivity to the individual models used in observing system design. Here, we give a brief overview of these evaluations and the open questions still remaining (or newly raised) by the current AMOC observing system.

### (a) Observing system experiments

In planning the RAPID $26^{\circ }N$ array, the approach was first to demonstrate in numerical simulations that the geostrophic method could work to capture the modelled AMOC variability. That is, using the output from an ocean model (velocity, seawater density), the sections at $26^{\circ }N$ in the model were subsampled as if a mooring were installed in the model. These subsampled variables were then used to compute the overturning transport using the thermal wind approach, etc., and then compared to the overturning transport computed from the output model velocities, i.e. model truth. Variations on the approach can be used to demonstrate how many and where moorings (or other ocean observations) are needed to observe the overturning transport [68–70].

In the case of the MOVE array at $16^{\circ }N$, the approach was similar [71,72] and demonstrated that in a model, the partial basin approach of MOVE was able to capture fluctuations of the southward transport at $16^{\circ }N$. More recently, the MOVE $16^{\circ }N$ and RAPID $26^{\circ }N$ methods were tested in a numerical simulation ($1^{\circ }$ and $0.1^{\circ }$ resolution), specifically addressing the question of how the reference level is chosen for the AMOC calculation [45].

As models have increased in resolution, similar tests can be performed after the observing system is already in the water. For example, Sinha *et al.* [67] re-evaluated the RAPID approach in a relatively high-resolution numerical simulation ($1/12^{\circ }$) and further developed a method to evaluate structural error in the methodology. This approach is valuable because it shows not just the first, fundamental result that an endpoint mooring approach is able to capture the AMOC transport variability, but further analysis can be used to refine observing methods to quantify and either reduce or accept residual error in the method.

In all cases, there may be slight variations due to model inaccuracies. For instance, if a particular numerical ocean model has strong southward flow in the deep eastern basin of the mid-Atlantic ridge, then the model approach to designing the AMOC might emphasize observations in the eastern basin. If the true ocean does not have these currents, then they might be unnecessary. Or, e.g. if the overturning transport in a numerical simulation is too shallow relative to the overturning in the true ocean, then it might de-emphasize other parts of the measurement system.

There is a bit of a chicken-and-egg problem: if we do not have an adequate observing system to know what the real ocean AMOC transport variability is, then we will not know that the model simulations used to design the observing system are inadequate. One often-accepted approach is to deploy more moorings initially to oversample regions where there is uncertainty, and then to subsequently remove the unnecessary ones from future deployments but with associated initial costs. It is not yet clear how to best combine incomplete observations with imperfect models to design an observing system.

### (b) Open questions

Some existing questions about the nature of the AMOC, and new questions raised on the basis of AMOC observations, remain unanswered. Key among these, which has become more obviously unclear with the installation of additional AMOC observing arrays, is the extent to which the AMOC behaves like a conveyor belt. This is a fundamental question of ‘what is the AMOC’ and what do we mean when we produce an AMOC observation at an individual latitude? Initially, our conceptual idea of the AMOC was based on the conveyor-belt idea which carries with it the misconception that when the circulation speeds up at one latitude, it speeds up by the same amount and simultaneously at all other latitudes. This is of course an oversimplification because it neglects the potential for water to turn or recirculate at a given latitude, but is also clearly not the view that one would derive from the AMOC transports provided by observing arrays (figure 2) which highlight the extensive high-frequency variability of transport estimates. Even so, the means of these time series are all roughly the same (17 Sv, [25]) which could indicate that after averaging over sufficiently long time periods, the AMOC is conveyor like.

What then do the high-frequency fluctuations mean, and what value do they provide to our understanding of the AMOC or its representation in numerical simulations? A future observing system which accounts for a basin-wide view of the AMOC could attempt to address these open topics:

— the extent to which the AMOC behaves as a coherent circulation or a single entity, rather than a stochastic collection of regional processes that when integrated and averaged produce a measurable quantity (the AMOC transport) – these two options are not mutually exclusive
— the relationship between the AMOC and convection or sinking, and between the AMOC and Greenland–Scotland ridge overflows [10,11,73]
— the near-term value of AMOC observations in either improving predictions or initializations of forecasting models [52]
— the extent of the cause–effect relationship between the AMOC and AMV [3,74]
— the importance (if any) to climate of high-frequency AMOC variability associated with mesoscale and smaller scale variability [75,76], or the degree to which resolving mesoscale processes is key to correctly simulating large-scale AMOC variability
— the utility of AMOC observations to identify and correct AMOC mechanisms/biases in numerical simulations
— the utility and/or accuracy of pre-instrumental proxy-based AMOC reconstructions, e.g. from sediment cores

The observations that provide time series of volume and heat transports should be supplemented by AMOC-related, process-based observations that can help the modelling community to evaluate how well their models represent those processes. As each observational program works independently to measure transports across individual sections, understanding meridional coherency of AMOC remains elusive. A coordinated effort among the observational programs, for example, could focus on if and how transport anomalies propagate across the observational sections. Here, an approach could be to use dye tracers to track water masses. Another question that needs to be confronted is whether we are currently measuring the AMOC at the relevant latitudes if we are interested in the buoyancy-driven, low-frequency variability that is thought to be important for climate vs whether we require measurements in regions where energetic small-scale variations and watermass changes are happening.

## 4. Future AMOC observing system

We have been asked to provide a perspective on whether AMOC observations should continue, how they should continue and why? In the aforementioned sections, we addressed the question of whether they should continue (yes) and why (myriad benefits of AMOC observations). Here, we now attempt to address the ‘how’ which is complicated. On the one hand, we have summarized the essential approaches used in AMOC observing arrays (§2c) and identified existing shortcomings (uncertainties §2d and open questions §3b). It would be possible to propose a continuation of existing observing arrays while addressing the shortcomings that infringe upon accuracy, and methodological differences that impede addressing open questions. This approach is logical. It is also true that maintaining existing arrays is the quickest way to gain the long time series of AMOC transports which are necessary for understanding low-frequency variability. If instead we substantially transform the observing approach, to the point of removing or moving observing arrays (i.e. losing continuity of measurements), we will have lost the ability to achieve a longer record sooner and would be potentially ‘starting over’. If we continue existing arrays, we will have a 40-year record by 2045; if we remove/move existing arrays and restart in 2025, we will need to wait until 2065 to have a 40-year record. At the same time, this is an insufficient argument for simply continuing the arrays as they currently stand. It does not recognize the opportunity cost of expending a great deal of resource (both financial and human) on the existing arrays vs another endeavour. It also does not let us maximally leverage the knowledge and experience we have gained from making AMOC observations for 20 years to ask the question ‘How now should we make AMOC observations?’

A few array/latitude specific questions to bear in mind while considering AMOC observing approaches that may move or remove existing arrays:

— What will we gain from $26^{\circ }N$ observations, where the longest-to-date records of AMOC volume, heat and freshwater transports are now available, but a substantial part of the variability is wind-driven and the observational limits may preclude capturing the buoyancy-forced (potentially smaller) part of the transport variability?
— What will we gain from OSNAP, spanning the northern high latitudes transformation region with the potential to unpick the buoyancy-forced sources of AMOC variability (Labrador Sea vs Greenland–Scotland ridge overflows vs surface transformation of the Atlantic inflow waters towards the Arctic)?
— What about $16^{\circ }N$ and $11^{\circ }S$ spanning the tropical Atlantic where, somehow, the AMOC transport passes across the equator suffering a change in sign of f while still maintaining a southward flowing deep western boundary current and net northward heat transport?
— SAMBA $34.5^{\circ }S$ measures at the southern gateway to the Atlantic, where stability of the AMOC has been defined from the sign of the salt transport across this gateway. What if our focus on the North Atlantic has caused us to neglect the South Atlantic which is the part of the Atlantic (and AMOC) that is unique compared to other ocean basins in that the net ocean heat transport in this region is equatorward?

Despite having arrays spanning a wide range of latitudes in the Atlantic, the observing system as it currently stands suffers from methodological differences that may impede our ability to compare transports between individual latitudes to answer the conceptual question of ‘what is the AMOC?’

### (a) Observing system design

**Figure 5.**
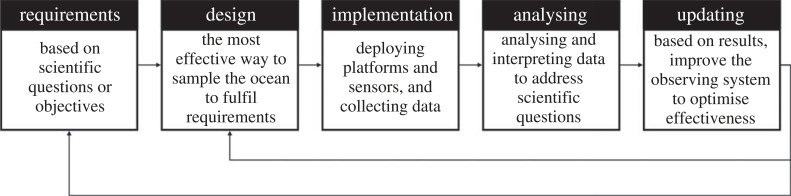
An iterative process for designing an ocean observing system.

An ocean observing system is a large-scale national, regional or international program founded and funded by governments and stakeholders with the goal of coordinating observing activities carried out by many partners within the region. The purpose of an observing system depends on scientific interests, government interests and societal needs. There are different ways to capture this information, but one such is in figure 5. There are roughly five steps in the process:

— Requirements definition: Based on the scientific questions and objectives that the observing system should address, what must the observing system capture/quantify?
— Design: Based on requirements, how can available measurement platforms and sensors be deployed to capture ocean variability at the required locations and times, and how should those data be processed to achieve outcomes? Data sharing protocols can also be outlined.
— Implementation: Deploy the measurement platforms and sensors, collect and process data.
— Analysis: Analyse and interpret the data to address the scientific questions. By carrying this through to the end goal, including error and uncertainty quantification.
— Update and improve: Based on results, the observing system can be improved and optimized to increase its effectiveness at delivering requirements.

In carrying out these steps, both observation- and model-based assessments should be leveraged. To date, this approach has been applied for individual arrays at specified latitudes. In table 2, the publications detailing the observing system design or evaluation are given for each array, which verified that the array was fit for the purpose of estimating the AMOC transport or its variability (i.e. anomalies from the mean) or heat or freshwater transports. With nearly two decades of AMOC observing experience, and several years of overlapping records between latitudes (figure 2), we are now in a position to apply this approach to an Atlantic-wide MOC observing system. Prescribing a future AMOC observing system is well beyond the scope of this article; here, we only aim to provide some guiding questions and directions to be considered by the community in taking a community-wide view to future AMOC observing needs.

**Table 2.** Assessments of transport arrays in the literature. The methodology column lists papers that describe the methodology. The evaluation column is the assessment of the method in calculating the AMOC and in some cases, the meridional heat and freshwater transports. Note that the calculation at $41^{\circ }N$ is not a mooring array, but rather a calculation based on Argo and altimetry data. We include it here as it provides also an observing system evaluation for an Argo-based approach.

array	methodology	evaluation
RAPID $26^{\circ }N$	Rayner *et al.* [77],	Hirschi *et al.* [68],
	Johns *et al.* [78],	Baehr *et al.* [69],
	McCarthy *et al.* [40],	Roberts *et al.* [79],
	McDonagh *et al.* [80]	Stepanov *et al.* [81],
		Sinha *et al.* [67],
		Danabasoglu *et al.* [45]
OSNAP	Lozier *et al.* [82],	Li *et al.* [24]
	Li *et al.* [24],	
	Li *et al.* [83]	
NOAC $47^{\circ }N$	Rhein *et al.* [84],	Breckenfelder *et al.* [85]
	Nowitzki *et al.* [86],	
	Wett *et al.* [87]	
SAMBA $34.5^{\circ }S$	Meinen *et al.* [88],	Baehr *et al.* [89],
	Meinen *et al.* [31],	Perez *et al.* [90]
	Kersalé *et al.* [91],	
	Kersalé *et al.* [32]	
TRACOS $11^{\circ }S$	Herrford *et al.* [36],	Baehr *et al.* [89],
	Tuchen *et al.* [92]	Herrford *et al.* [36]
MOVE $16^{\circ }N$	Kanzow *et al.* [71],	Kanzow *et al.* [93],
	Send *et al.* [44]	Danabasoglu *et al.* [45]
$41^{\circ }N$	Willis [66],	Willis [66],
	Hobbs & Willis [94]	Hobbs & Willis [94]

### (b) Defining the requirements

Initially, individual observing arrays set out to quantify the AMOC transports (either volume transport only, or volume transport anomaly only, or also including heat and freshwater transport estimates). More recently, the capability to address other property transports (nutrients and carbon) using the same infrastructure have come to light and been implemented [95–97]. Defining requirements for an Atlantic-wide MOC observing system will need consultation with stakeholders to identify: priorities for which observational parameters (measured or derived) should be provided, and what level of accuracy or precision is required; time resolution (whether monthly, seasonal or annual snapshots or averages are needed) and the latency or frequency in updates needed for different purposes. (i.e. Is there a requirement for near real-time estimates or estimates with 1 month or 1 year delay?); and the duration of observational records needed (e.g. based on the time required to identify anthropogenic causes of change).

Such requirements must be specified in order that the AMOC observations could be used to achieve scientific objectives such as:

— Understanding the dominant processes of AMOC variability across time scales from seasonal to millennial,
— Understanding the role the AMOC plays in the climate including redistributing heat (or other variables) in the Atlantic, setting or responding to atmospheric fluxes,
— Benchmarking numerical ocean, climate or forecasting models,
— Improving process-understanding both for fundamental understanding or improvements of process-representation in models.

This is only a short list of potential scientific objectives for an AMOC observing system; a more comprehensive list could be developed through stakeholder engagement (e.g. scientific community, ocean and climate modellers, operational forecasting centres, public stakeholders or other users of AMOC dataset). Prioritizing different requirements may be somewhat subjective, and should be designed as an iterative process. As greater understanding of the natural system is achieved, as uptake and use of AMOC observations become more widespread, as technological advances are realized, as the needs of stakeholder communities change—so too the requirements of an AMOC observing system may evolve. A critical aspect of a successful process is wide and effective community engagement, to ensure both primary and secondary uses of an AMOC observing system are considered, and to work towards community consensus on an agreed set of priorities.

### (c) Designing an observing strategy

Once we have a set of requirements, how do we decide what combination of *in situ* assets, remote sensing data sources and methodological approaches will deliver the required observations? Previously, the approach has been to use an OSE as described in §3a. By using this approach, we have a number of AMOC observing arrays that were designed using a numerical ocean simulation (usually a single model) and based on a methodological approach created for that latitude [68,69]. Observationally, one consequence of the single-latitude approach is that we have transport estimates that are not comparable, which precludes or at least complicates answering some open questions about meridional coherence of the AMOC or the relationship between transports between latitudes. Other methods may include adjoint approaches [98,99] or machine learning, or comparing a few latitudes [89,90], or tailoring individual approaches for a given latitude through subsampling exercises (i.e. If a mooring is removed from the array, what is the increase in uncertainty on the AMOC estimate and is the subsequent total uncertainty within tolerance?). In designing a full-Atlantic MOC observing system, it may be possible to expand the use of OSEs to evaluate a network of observing arrays, and how—if an individual array were removed or downscaled, or a new array added—the whole network would change. A full perspective on designing an AMOC observing strategy is beyond the scope of this article.

An additional consideration in the design of a system is the cost of the system, relative to the expected benefit. We do not attempt to carry out a cost–benefit analysis of AMOC observations, but rather to pose some questions to be considered by the community and the government stakeholders/funders. Using research vessels carries a financial cost (roughly £40 k±10 k/day for a global class vessel) and requires a crew to operate the vessel and a team of scientists and technicians to deploy the platforms and sensors. Roughly speaking, the cost of an AMOC observing array is on the order of £1 M/year for continuous AMOC observations, with substantial variances between latitudes depending on the complexity of the region. However, other observing methods also have associated costs (e.g. an Earth observation satellite on the order of £100M-1B). Putting a financial value on the benefit is not straightforward (to these authors), but perhaps relevant alternative costs to consider might be the cost of hurricane damage (roughly £2T between 1980 and 2021 [100] or the possible costs of a carbon scrubbing system ($500M/unit or $60/metric ton of ${\mathrm{CO}}_{2}$ [101]) to weigh up the relative benefit of natural carbon storage (roughly 155±31 PgC=155 billion tonnes [2]). It is not yet clear to what extent AMOC observations can improve, e.g. hurricane forecasts, nor the relationship between the AMOC and anthropogenic carbon storage in the ocean, nor the value to climate mitigation and adaptation strategies if better projections of regional climate change were possible. The calculations to relate the benefit of science against economic costs are not straightforward, but it is clear from the latest IPCC AR6 report [11] that our understanding of past and future AMOC variability still leaves significant questions open, including the likelihood of an AMOC shutdown by 2100.

### (d) Data accessibility

Before concluding, it is worth mentioning that the full benefits of an AMOC observing system cannot be realized without considering how data will be provided and accessed. At present, there is no agreed common format for AMOC transport observations. This means that for a non-expert user, including any member of the scientific community who is not part of a particular observing array team, additional effort is required to evaluate data from more than one array. Substantial progress in this direction has been made especially through the efforts of the OceanSITES group (oceansites.org), for instance in standardizing the file format (netCDF) with prescribed data structures for individual data types from moored observations. However, no one format for transports nor accessible (web) location has been agreed. Progress towards improving existing data accessibility can be made in parallel with and to inform future observing system design.

In considering future observing system design, a more complete adoption of FAIR principles should be part of the objectives [102]. In addition to common data formats which are machine readable and using a standard vocabulary to define variables, the programming scripts (code) used to make data products should also be made publicly available. This is an objective which will require substantial human effort on the part of caretakers of existing observing arrays, and should be a funded endeavour recognizing both the value and the effort required to accomplish the aims.

In addition, the present AMOC observing system has varying delays (latency) in data availability. There are unavoidable delays to updating transport time series from observing arrays, associated with the synchronicity (or lack thereof) of research cruises to service the moorings. Additional delays are associated with cost-saving measures (both financial and carbon) for ship-based expeditions: the moorings are left in the water for as long as possible before being serviced. In these cases, some strategies are available to retrieve data more frequently: moorings with surface expressions (e.g. floats at the sea surface) could telemeter data in near real-time but may add a multiplicative factor to the cost; *in situ* assets with the possibility for acoustic data retrieval (e.g. by gliders or autonomous surface vehicles) could achieve reductions in data latency at a lower cost.

To give an idea of the data latency, the RAPID $26^{\circ }N$ transport calculation requires moored data from both sides of the Atlantic which are serviced on separate research cruises separated by up to a year in time. At the time of writing (February 2023), the AMOC transport time series for $26^{\circ }N$ is available until December 2020, which is the last time the western boundary array was serviced. The eastern boundary array was serviced in February 2022, meaning that data from the eastern boundary array are available until February 2022; however, the western boundary data (at the time of writing) were still in the water from January 2021 through February 2023; as of February 2023, the latest date where data are available from both boundaries is then December 2020. After the servicing of the western boundary is concluded in March 2023, quality control and processing will be completed within 6 months (September 2023), at which point the time series can be updated through February 2022 (the new latest date when both boundary datasets are available). Table 3 illustrates the data latency for RAPID, accounting for differences in time of array servicing and latency to allow for data quality control and processing.

**Table 3.** Research expeditions to service the RAPID $26^{\circ }N$ array, and associated timing of transport estimates.

sub-array (boundary)	expedition start	expedition end	end date east and west	data release (end + 6 mo)	version
West	Feb 2023	March 2023	Feb 2022	Sep 2023${\;}^{a}$	v2022.1
East	Feb 2022	March 2022	Dec 2020	Aug 2022	v2020.2
West	Dec 2020	Jan 2021	March 2020	July 2020	v2020.1
East	March 2020	April 2020	Sep 2018	Aug 2020	v2018.2

${\;}^{a}$The expected data delivery date, after ∼6 months of quality control and processing. The table is given in reverse chronological order, where the top line represents the current activity on the array: the western boundary array is being serviced in February 2023 after which the time series can only be updated through March 2022 (end date) when the eastern boundary array was last serviced. Due to quality control and processing requirements, the time series through March 2022 will then be available 6 months after the current research expedition finishes (March 2023+6 months→September 2023).

## 5. Conclusion

Here, we have revisited our motivation for making AMOC observations, outlined some of the gains in understanding due to those observations, and the progress made so far in evaluating AMOC observing. We have further raised some questions to be addressed by the community in considering the future of an AMOC observing system which accounts for knowledge gained to-date, shortcomings of existing observing which impede our ability to answer fundamental questions about the AMOC, and how we might go about designing a future AMOC observing system as a community.

To conclude, we reiterate the gains in understanding that have been achieved due to direct observation of the Atlantic overturning transports: that the large-scale ocean circulation is variable on all observed time scales, and that the observations show larger amplitude variations than are simulated in coupled climate models. This primary finding challenges our previous conceptual understanding of the overturning circulation as a slowly varying circulation pattern, with fluctuations on decadal and longer time scales, where these fluctuations are primarily associated with either buoyancy forcing in higher latitudes (where the deep ocean is ventilated) or with the net effect of mixing on basin-scales driving widespread but slow upwelling.

The direct observations have further cemented the idea that the overturning transports are responsible for the northward heat transport spanning all latitudes in the Atlantic. However, with the proliferation of observing arrays at a range of latitudes, these continuous observations have called into question the notion of the AMOC as a ‘conveyor belt’ where observations at a single latitude can be used to understand the Atlantic-wide phenomenon of overturning. While on long time scales (the observed mean AMOC transport) appears to have a consistent value across all latitudes (about 17 Sv), the shorter-time scale fluctuations show no apparent coherence of transport fluctuations. It is not clear at this point whether observed differences are due to methodological differences in measuring transports or represent the true nature of the Atlantic circulation—a point that must be addressed by the community with urgency.

When considering the future of AMOC observations, it is clear that the gains achieved would not have been possible without direct observations. Looking into the future, we will need a considered approach for how to maintain measurements of this climatically critical circulation pattern. The future benefits cannot be understated. Observations are needed to understand the consequences of global warming on the large-scale ocean circulation which has been confirmed as responsible for heat, freshwater, nutrient and carbon redistribution in the Atlantic. These observations provide an integral measure of the circulation which is needed by the climate modelling communities to validate and verify that the representation of the ocean is adequate in present-day simulations and thus may be a trustworthy source of information about the future evolution of climate. The approach towards future observations requires that identified key uncertainties in observational methods be addressed (§2d) and that we use the best tools now available to quantitatively evaluate observing solutions. No single institution, AMOC observation record or observing system experiment in a single ocean model can be used to answer these questions; a community approach is required to achieve consensus in requirements and an observing solution which is coordinated over the whole Atlantic basin.

## Data Availability

This article has no additional data.

## Footnotes

1 For the RAPID/MOCHA/WBTS array [28], hereafter RAPID $26^{\circ }N$, initially the geostrophic calculation was performed between moorings on the western boundary of the basin (at $26.5^{\circ }$) and the mid-Atlantic ridge (MAR, at $24.5^{\circ }N$), and between the MAR and eastern boundary (around $27.5^{\circ }N$). In each of the piecewise calculations, an average latitude was used to choose a constant f. However, in 2020, the MAR moorings were removed and the geostrophic calculation is now performed between the western boundary moorings and the eastern boundary moorings (RAPID team, personal communication). This results in a different average latitude for f, which affects the computed strength of the overturning circulation.

2 One potential option to reduce the effect of this choice on comparisons between latitudes would be to use an AMOC-Ekman quantity. The method to compute this would still need to be agreed, and it could introduce new issues. For example, at $26^{\circ }N$, the Ekman transport is computed from ERA5 winds. The baroclinic transport variability derived from dynamic height also includes the ocean response to Ekman transport—which at $26^{\circ }N$ manifests as isopycnal displacements between 2000 and 3000 m [37]. If AMOC-Ekman were considered, these baroclinic signals would still be present in the computed transport variability but without being balanced by the Ekman transport.
